# The influence of caregiver depression on adolescent mental health outcomes: findings from refugee settlements in Uganda

**DOI:** 10.1186/s12888-017-1566-x

**Published:** 2017-12-19

**Authors:** Sarah R Meyer, Mara Steinhaus, Clare Bangirana, Patrick Onyango-Mangen, Lindsay Stark

**Affiliations:** 1 Program on Forced Migration and Health, Mailman School of Public Health, Columbia University, 60 Haven Avenue, New York City, NY 10032 USA; 2 International Center for Research on Women, New York City, USA; 3 TPO Uganda, Plot 3271 Kansanga, Opp. KIU, 21646 Kampala, Uganda

**Keywords:** Mental health, Depression, Anxiety, Violence, Refugees

## Abstract

**Background:**

Family-level predictors, including caregiver depression, are considered important influences on adolescent mental health. Adolescent depression and anxiety in refugee settings is known to be a significant public health concern, yet there is very limited literature from humanitarian settings focusing on the relationship between caregiver mental health and adolescent mental health. In the context of a larger study on child protection outcomes in refugee settings, researchers explored the relationship between caregiver depression and adolescent mental health in two refugee settlements, Kiryandongo and Adjumani, in Uganda.

**Methods:**

Adolescents between 13 and 17 and their caregivers participated in a household survey, which included measures of adolescent anxiety and depression, and caregiver depression. Analysis was conducted using multiple logistic regression models, and results were reported for the full sample and for each site separately.

**Results:**

In Kiryandongo, a one-unit increase in a caregiver’s depression score tripled the odds that the adolescent would have high levels of anxiety symptoms (AOR: 3.0, 95% CI: 1.4, 6.1), while in Adjumani, caregiver depression did not remain significant in the final model. Caregiver depression, gender and exposure to violence were all associated with higher symptoms of adolescent depression in both sites and the full sample, for example, a one unit increase in caregiver depression more than tripled the odds of higher levels of symptoms of adolescent depression (AOR: 3.6, 95% CI: 2.0, 6.2). Caregiver depression is a consistently significantly associated with adverse mental health outcomes for adolescents in this study.

**Conclusions:**

Adolescent well-being is significantly affected by caregiver mental health in this refugee context. Child protection interventions in humanitarian contexts do not adequately address the influence of caregivers’ mental health, and there are opportunities to integrate child protection programming with prevention and treatment of caregivers’ mental health symptoms.

**Electronic supplementary material:**

The online version of this article (10.1186/s12888-017-1566-x) contains supplementary material, which is available to authorized users.

## Background

Children and youth living in refugee camps face a series of significant risk factors for adverse mental health outcomes [1, 2]. There is extensive literature documenting a range of these risk factors, with a focus on exposure to traumatic events most prominent in the literature. For example, a systematic review of risk and protective factors for children affected by conflict found that “[e]xposure to violence is the factor with the strongest evidence base for the risk of subsequent psychological disturbances,” noting that evidence has shown that “[t]he degree of direct exposure to threat, cumulative number of adverse events, and duration of exposure, all consistently increased the odds of mental health symptoms” [1].

Recent research has also identified a number of household-level factors that influence children’s mental health in refugee settings, including mental health and well-being of caregivers [3, 4]. In refugee camps, caregivers may be exposed to sexual and gender-based violence, bereavement due to loss of family and community members, and changes in socio-economic status, all of which may be risk factors for elevated symptoms of mental distress [5]. Caregiver mental health may impact child and adolescent well-being in a range of ways. For example, caregivers with high levels of distress may be more likely to engage in substance abuse, violent behaviour, or harsh parenting and discipline, which can result in a household environment with increased risk of abuse or neglect [6].

There is limited literature from humanitarian settings focusing on the influence of caregiver mental health on child and adolescent mental health and well-being. A longitudinal study of former child soldiers and their caregivers in Sierra Leone, improved caregiver mental health was associated with improved mental health of youth [7]. In a study of Eritrean refugees in a refugee camp in Ethiopia, caregiver distress was a robust predictor of adolescent internalizing and externalizing emotional and behavioral symptoms [8]. Several other studies investigating this relationship amongst refugees have focused on refugee populations who have immigrated to high-income settings leaving a gap in the evidence-base concerning refugee children still residing in low and middle-income settings, particularly in refugee camps [9].

A systematic review of risk and protective factors for displaced and refugee children’s psychological health in low and middle-income settings noted the gaps in literature regarding parental mental health and children’s mental health, finding:

*Parental wellbeing has also received minimum attention, yet…. evidence from war-affected and non-refugee populations indicates this type of interaction is likely to be a central factor in children’s psychological health* [1]*.*

Psychological literature indicates that improvements in parental depression can result in improvement in children’s well-being and functioning [10].

Outside humanitarian contexts, and in the broader psychological and public health literature, caregiver mental health as a predictor of adolescent mental health, is also underexplored. Some analyses include children up to the age of 17 [11], however, there is a primary focus on impacts of caregiver mental health on children of younger ages [12]. Adolescent mental health has been recognized as a global public health challenge, with adverse mental health outcomes strongly associated with a range of risk-behaviors and poor outcomes across the lifecourse [13]. The role of genetic influences on adolescent mental health outcomes is established, and there is a strong focus in the literature on parent-adolescent relationships [14], however, there is a weaker evidence base on the specific role of caregiver mental health. Some investigations have explored the role of family structure and family functioning [15], caregiver depression as a mediator of the relationship between stressful life events and adolescent depression [16] and general risky family environments [17] as influences on adolescent mental health. However, epidemiological and intervention evidence, as well as hypotheses concerning mechanisms of influence, regarding caregiver mental health and younger children is significantly more developed than that focused on adolescents.

It is evident that further understanding of the predictors of adolescent mental health in humanitarian settings is needed, and such that caregiver mental health may be an important influence, further investigation is warranted. The present study seeks to contribute to the evidence-base concerning the relationship between caregiver mental health and adolescent well-being in humanitarian settings, examining this relationship in the context of two refugee settlements in Uganda, and focusing on an often over-looked population group, adolescents.

## Methods

The specific objectives of this study are to assess the influence of caregiver mental health on symptoms of depression and anxiety amongst adolescent South Sudanese refugees in Kiryandongo and Adjumani refugee settlements, Uganda. The research questions are: i) Is caregiver depression associated with higher levels of adolescent anxiety and depression in a sample of adolescent refugees from South Sudan, and ii) Do these associations differ according to outcome (depression or anxiety) or site (Kiryandongo and Adjumani). This study is embedded within the context of a larger project, “Measuring Impact though a Child Protection Index,” a study that seeks to understand the influence of child protection system strength on child protection outcomes, including exposure to violence and psychosocial well-being, amongst adolescent refugees.

### Study sites and sampling

At the time of the study, there were 102,237 refugees from South Sudan residing in Adjumani (UNHCR, 2015) and 35,177 in Kiryandongo (UNHCR, 2014). In both settlements, the majority of refugees from South Sudan had fled and arrived in Uganda after December 2013, when an outbreak of violence in Juba led to movement of a large number of South Sudanese to neighboring countries, including Uganda. Both Kiryandongo and Adjumani were considered active emergency settings at the time of data collection, and are sites where TPO Uganda, the primary research partner, has ongoing interventions.

Inclusion criteria for adolescents were that the respondent was between 13 and 17 years of age, was South Sudanese, and provided informed assent to participate in the interview. Caregivers, identified as the primary caregiver, provided informed consent for their own interview, as well as consenting for the adolescent to participate in the study. Adolescents were asked to identify a primary caregiver, and were given the definition of someone who “provides food, clothing, and any other basic needs and also provides emotional care.” While some caregivers may be biological parents of adolescent respondents, a significant proportion had non-parental or non-biological relationships with the adolescent respondents.

The study employed systematic random sampling in both refugee settlements. The sampling frames available from international agencies did not accurately reflect household numbers and population size in either refugee settlement, given the rapidly changing nature of the refugee population. A mapping exercise was conducted in both settlements, to develop a more complete sampling frame, and this enabled selection of a sampling interval based on the number of households, target sample size, expected prevalence of households with eligible adolescent respondents and expected refusal rate. The total sample size of randomly selected households in Kiryandongo was 220 households and in Adjumani was 251 households. Seven other respondents were dropped from analysis, as they were not South Sudanese and therefore did not fit the inclusion criteria, and one respondents was below the age of 13, resulting in a total sample size of 463 households for this analysis.

### Interviewer training

Data collectors were refugees who were recruited from within the refugee settlements, so as to ensure proficiency in required languages. Twelve data collectors were recruited in Adjumani and 13 in Kiryandongo, and all data collectors were individuals who had previously worked with TPO Uganda, providing community outreach or leading psycho-education sessions. All data collectors participated in an eight-day training, including a focus on all ethics procedures. Data collectors were provided with specific training on confidentiality, recognizing and addressing protection risks, and providing referrals and support. In addition, data collectors participated in extensive role play focusing on issues that could arise in the field, including participants not understanding questions, and were provided with daily supervision regarding appropriate response to participants’ questions. In Kiryandongo, two members of the team were selected as supervisors, who checked data quality and provided feedback to other members of the team, and these supervisors traveled to Adjumani to oversee data collection to ensure continuity and consistency of data collectors’ work.

### Data collection procedures

Upon entering a selected household, the data collectors identified the primary caregiver, in order to provide a short introduction to the study and obtain permission to interview an adolescent aged between 13 and 17. The data collector then sought informed consent from the caregiver, to participate in the caregiver survey, and then subsequently sought informed consent from the adolescent, to complete to adolescent survey. The adolescent survey was only conducted if a caregiver gave permission. Informed consent included description of the purpose of the study, that the information that the adolescent or caregiver shared would be confidential, that the participant would not be compensated, that the participant would not directly benefit from participation in the study, and that refusing to participate would not impact their access to services in the refugee settlements.

Data collectors ensured that the interview took place in a private setting, to protect confidentiality and enable respondents to feel comfortable responding to sensitive questions. Respondents were given a list of services in the settlement that they could access if they wished. Given the need for confidentiality, adolescents and caregivers who reported high levels of adverse mental health symptoms or exposure to violence were not automatically referred to services, but were encouraged to access existing services through provision of the aforementioned list of services. All interviews with female adolescents were conducted by female data collectors given the sensitive nature of some questions.

### Instruments

Instruments were translated into the two languages most widely spoken in these refugee settlements, Dinka and Nuer. The instruments were developed in English and translated initially by a refugee who was not a member of the research team. The first translation was then reviewed by the group of data collectors, and further refined during pilot testing of the instruments, which included cognitive interviewing of a sample of participants who did not participate in the full study. Surveys were conducted in Dinka and Nuer in Kiryandongo, and only Dinka in Adjumani (as the selected site within the settlement, Ayilo I, was populated with only Dinka speakers).

### Adolescent instrument

#### Demographics

Adolescents were asked about several demographic characteristics, including school attendance, educational attainment, and living status with regards to their parent or caregiver (Additional File 1).

#### Violence

Adolescents’ exposure to violence and abuse was assessed using questions adapted from studies of Violence against Children designed by the Centers for Diseases Control and the ISPCAN Child Abuse Screening Tool – Children’s Version [18, 19]. Adolescents were asked about seven types of physical abuse, six types of verbal abuse, and two types of sexual abuse. Adolescents were considered to have experienced a form of violence (physical, verbal, or sexual) if they answered yes to any of the questions asked about that form of violence. Then, types of violence were aggregated into one measure by summing the number of these three types of violence an adolescent experienced into a scale from zero to a possible three.

#### Anxiety

Levels of anxiety among adolescent participants were assessed using the 5-item version of the Screen for Child Anxiety Related Disorders (SCARED), assessing anxiety symptoms experienced in the past 3 months, with a 3-point scale (range 0–10) [20]. Means were imputed for missing values of those participants who refused one question of the scale; no participants refused more than one. The Cronbach’s alpha for this scale was 0.79. Scale scores were transformed into a binary score of “high” levels of symptoms of anxiety (versus not high) using a cutoff score of four and above as “high” [21].

#### Depression

Symptoms of depression within the past 2 weeks among adolescent participants were assessed using the Mood and Feelings Questionnaire Child Self Report (MFQ-C), short version [22]. The original scale consists of 13 questions scored on a scale of zero to two with a total score range from zero to 26. Means were imputed for missing values of those participants who refused three or fewer questions of the scale; no participants refused more than three. The Cronbach’s alpha for this scale was 0.88. Scale scores were transformed into a binary score of “high” levels of symptoms of depression (versus not high) using a cutoff score of 10 and above as “high” [23].

As indicated, in the case of both depression and anxiety outcomes for adolescents, a cut-off was selected and the outcome measure assessed as a categorical variable, in order to enable identification of individuals with higher levels of symptoms. The cut-off selected does not represent a diagnosis of a mental health condition, and was selected based on psychometric studies of these scales.

### Caregiver instrument

#### Depression

Symptoms of depression among caregivers were measured using the Hopkins Symptom Checklist-25 (HSCL-25) [24]. The original depression sub-scale consists of 15 questions, scored on a scale of one to four, with one being “Never,” two being “Sometimes,” three being “often,” and four being “All the time.” Poor translation of two questions (“how often have you cried easily,” and “how often have you felt worthless”) necessitated them being dropped from the scale scoring. In addition, the question “In the past week, how often have you had thoughts of ending your life?,” was removed, as mental health providers in the settlement did not have capacity for referrals. This resulted in a final scale consisting of a total of 12 questions. Means for single questions were imputed for eight participants who refused one question each. The Cronbach’s alpha for this scale was 0.81 (Additional File 2).

#### Socioeconomic status

**Fig. 1:**
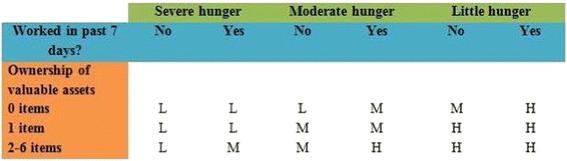
Categories of caregiver socioeconomic status

Socioeconomic status was determined by combining information on household hunger, caregiver employment status, and the number of valuable assets in the household. Household hunger was determined using the FANTA Household Hunger Scale, which categorizes households into one of three categories: “little to no hunger,” “moderate hunger,” and “severe hunger” [25]. Caregiver employment was considered binary based on a yes or no response to the question “Have you worked in the past seven days?” Possession of valuable assets was determined by caregiver’s report of whether or not any member of their household owned each of five valuable assets (watch, bicycle, cell phone, radio, livestock/herds/poultry). Caregivers were then categorized into three socio-economic statuses, low, medium or high, as illustrated in Fig. 1:

### Statistical analysis

All data were collected using mobile phone based platforms, and exported from a secure server into Stata 12.1 for all analyses. Analyses were conducted adjusting for survey design, with each settlement included as strata, and combined and subpop regressions conducted. Associations between all variables were examined using pairwise correlation. Multiple logistic regression models were tested, with a full model including adolescent gender (coded 0 for male, 1 for female), adolescent age, socio-economic status (low = reference, compared to medium or high), exposure to violence (how many types of violence experienced out of a possible three), caregiver depression (measured as a continuous variable), and whether or not the adolescent was living with at least one biological parent (binary variable). Regressions were performed both on the combined sample of sites and within each site individually, with the following variables included as potential confounders: adolescent’s age, caregiver socio-economic status, gender, whether the adolescent was living with a biological parent or not, and exposure to violence. Confounders were selected for inclusion in the models based on the strong body of literature identifying these variables as potential confounders in high-income settings, and the emerging evidence concerning humanitarian contexts. Sensitivity analyses were conducted, running multiple logistic regressions of depression and anxiety outcomes, utilizing a cut-off one point below and one-point above the cut-offs used in this analysis. These analyses indicated findings consistent with the results using the cut-off points utilized in this analysis.

### Ethical approval

The research was approved under the Columbia University Medical Center IRB AAAB7134. In Uganda, the researchers obtained permission to conduct research in the refugee settlements from the Office of the Prime Minister. Research partners, including TPO Uganda, who has operated services in the refugee settlements for two decades, provided significant insight into the appropriate ethical procedures, including referral structures, to ensure the safety and wellbeing of participants.

## Results

Table 1 displays descriptive statistics for all variables used in the analysis. In Kiryandongo, 51.4% of the adolescents sampled were male, compared to 53.9% in Adjumani. The average age of the sample was 14.7 years in Kiryandongo, ranging from 13 to 17, and 14.5 in Adjumani, ranging from 13 to 17. There was no evidence of a statistical difference in the gender or age composition of the sample between the two sites. There were, however, statistically significant differences between the two sites for all remaining variables.

**Table 1:** Descriptive statistics, by site

	Kiryandongo *N* = 218						Adjumani *N* = 245						Difference between sites
	*n*	%	Mean	Std. Dev.	Min	Max	*n*	%	Mean	Std. Dev.	Min	Max	p
Adolescent’s gender													0.591
Male	112	51.4					132	53.9					
Female	106	48.6					113	46.1					
Household’s socio-economic status ^a^													**0.001**
Low	33	15.1					18	7.4					
Medium	106	48.6					101	41.6					
High	79	36.2					124	51.0					
Adolescent is currently living with at least one biological parent	103	47.3					186	75.9					**0.000**
Adolescent has high levels of symptoms of anxiety disorder (SCARED > = 4)	109	50.0					31	12.7					**0.000**
Adolescent has high levels of symptoms of depression (MFQ > = 10)	95	43.6					76	31.0					**0.005**
Adolescent’s age	218		14.7	1.5	13	17	246		14.5	1.4	13	17	0.129
Adolescent’s exposure to violence – number of types^b^	218		0.8	0.9	0	3	231		0.6	0.8	0	3	**0.031**
Caregiver depression score (HSCL-25)^c^	218		2.1	0.5	1	3.7	246		1.8	0.4	1	4	**0.000**

^a^Two missing in Adjumani

^b^15 missing in Adjumani

^c^Averaged over 12 of 14 standard questions, two dropped due to poor phrasing

There were also significant differences between the two settlements in symptoms of depression and anxiety amongst adolescents and caregiver depression scores. The prevalence of high levels of symptoms of an anxiety disorder among adolescents in this sample was 50.0% in Kiryandongo, compared to 12.7% in Adjumani, and the prevalence of high levels of symptoms of depression was 43.6% in Kiryandongo and 31.0% in Adjumani. The average adolescent in both sites had been exposed to less than one out of three types of violence—verbal abuse, physical abuse, and/or sexual abuse. Caregiver’s depression scores averaged 2.1 on a scale from 1 to 4 in Kiryandongo and 1.8 in Adjumani.

Table 2 presents odds ratios from logistic regression models for the outcome of high levels of symptoms of anxiety among adolescents. The results provide evidence that the factors associated with adolescent anxiety vary by site. In the model for the full sample, higher caregiver depression (AOR: 3.7, 95% CI: 2.1–6.8), being female (AOR: 3.1, 95% CI: 2.0, 4.9), and greater exposure to violence (AOR: 1.6, 95% CI: 1.2, 2.0) were all significantly associated with increased symptoms of adolescent anxiety, while living with a biological parent was significantly associated with decreased symptoms of adolescent anxiety (AOR: 0.5, 95% CI: 0.3, 0.8). In Kiryandongo, a one-unit increase in a caregiver’s depression score tripled the odds that the adolescent would have high levels of anxiety symptoms (AOR: 3.0, 95% CI: 1.4, 6.1) and being female more than quadrupled those odds (AOR: 4.5, 95% CI: 2.2, 9.0). In Adjumani, living with a biological parent reduced the odds of adolescents’ high levels of anxiety by 70% (AOR: 0.3, 95% CI: 0.1, 0.8). The only variable that was significantly associated with adolescent anxiety across both sites was exposure to violence. Adolescents who experienced one additional form of violence in Kiryandongo were 1.5 times more likely to report high levels of symptoms of anxiety (95% CI: 1.0, 2.1), while those in Adjumani were 2.6 times more likely to report high levels of symptoms of anxiety (95% CI: 1.5, 4.5).

**Table 2:** Odds ratios for logistic regression of adolescent symptoms of anxiety, combined and individual sites

	Combined *N* = 463		Kiryandongo *N* = 218		Adjumani *N* = 245	
	OR	95% CI	OR	95% CI	OR	95% CI
Caregiver depression score	***3.7	[2.1,6.8]	**3.0	[1.4,6.1]	2.6	[1.0,6.8]
Adolescent’s age	1.0	[0.8,1.1]	0.9	[0.8,1.2]	1.1	[0.8,1.5]
SES - Low	–	–	–	–	–	–
Medium	0.9	[0.4,1.9]	0.9	[0.4,2.2]	1.3	[0.2,8.2]
High	0.6	[0.3,1.3]	0.6	[0.3,1.5]	1.7	[0.3,10.4]
Gender	***3.1	[2.0,4.9]	***4.5	[2.2,9.0]	1.0	[0.4,2.6]
Living with biological parent(s)	**0.5	[0.3,0.8]	1.0	[0.6,1.9]	*0.3	[0.1,0.8]
Exposure to violence	***1.6	[1.2,2.0]	*1.5	[1.0,2.1]	**2.6	[1.5,4.5]
*Model characteristics*						
*N*		*446*		*218*		*228*
*Probability*		*0.0000*		*0.0000*		*0.0078*

* < 0.05; ** < 0.01; *** < 0.001

Table 3 presents odds ratios from logistic regression models for the outcome of high levels of symptoms of depression among adolescents. In contrast to the findings related to the outcome of adolescent anxiety, the results for the outcome of adolescents’ symptoms of depression are fairly consistent between the two sites and in the combined sample. In the combined sample, higher levels of caregiver depression (AOR: 3.6, 95% CI: 2.0, 6.2), being female (AOR: 2.7, 95% CI: 1.7, 4.1), and exposure to more forms of violence (AOR: 1.7, 95% CI: 1.4, 2.2) were all associated with higher levels of symptoms of adolescent depression. In Kiryandongo, the same three predictors were significant in the final model. In Kiryandongo, a one-unit increase in caregivers’ depression scores was associated with three times higher odds of adolescents reporting high levels of symptoms of depression (AOR: 3.0, 95% CI: 1.5, 6.1), female adolescents had nearly six times higher odds of reporting high levels of symptoms of depression (AOR: 5.6, 95% CI: 2.8, 11.4), and exposure to one additional form of violence doubled the odds of reporting high levels of symptoms of depression (AOR: 2.0, 95% CI: 1.4, 2.9). In Adjumani, a one-unit increase in a caregiver’s depression score was associated with four-times higher odds of adolescents reporting higher levels of symptoms of depression (AOR: 4.3, 95% CI: 1.5, 12.4), and exposure to one additional form of violence was associated with 80% higher odds of adolescents reporting higher levels of symptoms of depression (AOR: 1.8, 95% CI: 1.2, 2.6).

**Table 3:** Odds ratios for logistic regression of adolescent symptoms of depression, combined and individual sites

	Combined *N* = 463		Kiryandongo *N* = 218		Adjumani *N* = 245	
	OR	95% CI	OR	95% CI	OR	95% CI
Caregiver depression score	***3.6	[2.0,6.2]	**3.0	[1.5,6.1]	**4.3	[1.5,12.4]
Adolescent’s age	1.0	[0.9,1.2]	1.0	[0.8,1.3]	1.1	[0.9,1.4]
SES - Low	–	–	–	–	–	–
Medium	0.6	[0.3,1.1]	1.0	[0.4,2.5]	**0.1	[0.0,0.5]
High	0.6	[0.3,1.2]	0.9	[0.3,2.2]	*0.2	[0.1,0.8]
Gender	***2.7	[1.7,4.1]	***5.6	[2.8,11.4]	1.3	[0.7,2.5]
Living with biological parent(s)	0.7	[0.5,1.1]	0.6	[0.3,1.2]	1.3	[0.7,2.5]
Exposure to violence	***1.7	[1.4,2.2]	***2.0	[1.4,2.9]	**1.8	[1.2,2.6]
Model characteristics						
*N*		*446*		*218*		*228*
Probability		*0.0000*		*0.0000*		*0.0002*

* < 0.05; ** < 0.01; *** < 0.001

## Discussion

Adolescent refugees face a multiplicity of risks in refugee camp settings, and recent research has emphasized that these risks may result from household-level factors. Research has shown an “interplay of multilevel stressors” associated with adverse mental health outcomes for children affected by conflict; violence in the household is one such stressor, and caregiver mental health, though less well-understood, is emerging as an important influence on adolescent mental health in humanitarian settings [26–28].

There is some evidence from high-income settings regarding the potentially long-lasting influence of caregiver mental health on adolescent well-being [12]. A study of depressed parents showed high levels of reporting of behavioural problems amongst children of depressed parents, as well as symptoms including loss of appetite and difficulty sleeping [29]. A meta-analysis of 33 studies found that children of depressed mothers have significantly more conduct and behavioural problems [30]. These data, combined with increasing understanding of the role of family-level factors in determining adolescent mental health outcomes in humanitarian settings, indicate the need to understand and investigate this relationship in humanitarian contexts. The associations found in this study may be explained by several mechanisms that have been identified in broader literature [31]. Studies of the impact of parenting programs on parental psychological well-being indicate that one potential mechanism through which parental distress impacts adolescent psychological well-being is through parenting behaviors and marital relationships [12]. Another possible mechanism is that exposure to parental distress increases sensitivity to stressful life events in adolescents, thus increasing symptoms of adverse mental health outcomes [16]. The research literature indicates that the findings in this study show a credible relationship between caregiver mental health as a predictor of adolescent mental health.

This study of the association between caregiver mental health and adolescent anxiety and depression symptoms in two refugee settlements in Uganda indicates variations in the influence of caregiver mental health, by site and by mental health outcome. Caregiver mental health was strongly associated with adolescent symptoms of anxiety for the full sample and in Kiryandongo, controlling for socio-demographic variables and exposure to violence, while caregiver mental health was significantly associated with adolescent report of depression across the full sample and both sites. High odds ratios – for example, a one unit increase in caregiver depression being associated with more than four times the odds of an adolescent reporting high symptoms of anxiety – indicate the significant role of caregiver mental health in adolescents’ psychosocial well-being in this context. The variation in association between caregiver mental health and anxiety in Kiryandongo and Adjumani may be due to different household dynamics in Adjumani; in particular, at the time of the study, caregivers were reported to spend significant amounts of time traveling to South Sudan and back to Adjumani, whereas there was less movement of caregivers from Kiryandongo. The variation in influence of caregiver mental health on adolescent anxiety may be hypothesized to be at least partially explained by this difference.

Some other notable findings from this analysis indicate the strength of the comparative approach of this study, allowing insights into factors that may have particular importance or relevance in each refugee settlement. For example, gender was significantly associated with both depression and anxiety symptoms in Kiryandongo, and not in Adjumani, indicating the need to assess environmental factors that may result in increased adverse mental health outcomes for female adolescents in Kiryandongo. In addition, whether an adolescent lived with one or both biological parents was included as a potential protective factor; this was found to be protective for anxiety in the full sample and in Adjumani, but not for depression. The lack of a consistent pattern for this variable indicates a need to more fully understand adolescent living situations in these refugee settlements.

This study confirms the important influence of caregiver mental health on adolescent well-being in two refugee settlements in Uganda. The interrelationship between caregiver mental health and adolescent well-being is widely accepted in research literature, with a shift away from a primary focus on the role of conflict-related trauma exposure on mental health outcomes and a widening scope to assess family and household-level factors in humanitarian settings [4]. This study confirms and extends the existing literature on caregiver mental health and adolescent mental health in humanitarian settings; overall, the findings in the present study are consistent with the existing evidence concerning the influence of caregiver mental health on adolescent well-being in refugee settings [4, 7]. This study, and other evidence, indicates a role for interventions to improve caregivers’ mental health as a way to both promote caregivers’ well-being, as well as address adolescents’ mental health. A systematic review of parenting programs to improve parental psychosocial well-being in high-income settings notes that interventions to address parental psychological health are vital in order to “reduce the disruption to the child’s emotional, educational and social adjustment, and thereby to promote the mental health of future generations” [12]. Humanitarian agencies and donors should explore opportunities to test and implement interventions assessing the impact of mental health interventions on both caregivers and adolescents.

This sub-study was conducted in the context of a study of child protection, and the findings are considered here through a child protection lens. Caregiver mental health may significantly affect parenting, child-parent interactions and use of violence within the household; existing literature indicates that caregiver well-being may directly impact caregivers’ capacity to provide protection and care for children, and high levels of distress within a household may lead to increased child protection risks [32]. For example, a study in Northern Uganda showed that female caregivers’ history of childhood abuse, and male caregivers’ post-traumatic stress symptoms and alcohol-use, were significantly associated with self-reported use of violence against children [33]. In humanitarian settings, specifically, multiple and overlapping stressors, such as lack of access to livelihoods and caregivers’ own histories of exposure to traumatic events and conflict (for example, in the case of Kiryandongo and Adjumani, many caregivers were themselves refugees as children), significantly influence the socio-ecological context for adolescents, influencing risk and resilience factors vital for long-term health and development. Child protection policies and programs are designed to prevent harms against children and improve children’s well-being in humanitarian settings, yet key policy frameworks and approaches do not adequately address the influence of caregivers’ mental health [34]. These data indicate the centrality of caregiver well-being in adolescent mental health and therefore an opportunity to integrate child protection programming with prevention and treatment of caregivers’ mental health symptoms. In addition, parenting programs have been explored as an approach to improving household resilience and increasing safety and well-being for children; some parenting programs implemented in humanitarian contexts have shown promising results in terms of reduction of violence against children and improved child-parent interactions, and the potential for such programs to be integrated with treatment of caregivers’ symptoms of mental health and other child protection programming should be explored [35, 36]. Research indicating the role of family-level factors in influencing child and adolescent mental health outcomes has not adequately influenced intervention design and delivery in humanitarian settings [37].

Some limitations of this study include the utilization of a single measure of caregiver mental health. The type of violence experienced may have differential impacts on adverse mental health outcomes, therefore the use of a sum score for violence may influence the results. Patterns and associations between types of violence and psychosocial well-being using the same data are examined in a separate publication [38]. The adolescent anxiety and depression scales, and caregiver depression scale, have not been previously validated with South Sudanese populations; the researchers piloted the instruments and conducted cognitive interviewing to ensure the questions were understood and appropriate for the participants, however, a full validation study was beyond the resources of the present study. Mental health concepts were difficult to capture in Dinka and Nuer languages, particularly psychosomatic symptoms, and therefore translation accuracy may be a limitation. In addition, the cross-sectional nature of the study implies that temporality cannot be established and that the causal relationship posited in this analysis – that increased symptoms of caregiver depression influence adolescent depression and anxiety – cannot be established using these data alone. There is evidence in the literature concerning the bidirectional relationship of child and caregiver mental health [39, 40], and in particular, parenting and child mental health [41, 42]. Adolescent mental health may impact caregiver mental health, however, the temporal association posited in this analysis is supported by extensive literature. Future research could address the limitations of a cross-sectional study design, and there is widespread recognition that longitudinal research is needed to further understanding of the associations between caregiver and adolescent well-being in humanitarian contexts [43]. In addition, measures of family functioning, parenting styles and parental-report of adolescent mental health symptoms could have added the potential to explore moderators and mediators. Given the need to implement the survey instrument to both caregivers and adolescents in a short time period, researchers were unable to include these additional variables. Future research in this context could explore the interplay between caregiver mental health, other household-level predictors, and adolescents’ psychosocial outcomes.

## Conclusions

Adolescent refugees face significant risks to their mental health and well-being in refugee settings globally, and amongst these risks, caregiver mental health is poorly understood and under-researched. These findings add to an emerging body of literature that recognizes the importance of assessing the relationship between caregiver mental health and adolescent well-being; translating this understanding into effective policy and programming to protect adolescents and promote their health and well-being is also needed.

## Additional files

Additional file 1: Adol final instrument. Adolescent quantitative assessment. Measures of socio-demographics, violence, psychosocial well-being and social support. (DOCX 113 kb)

Additional file 2: Caregiver final instrument. Caregiver quantitative assessment. Measures of socio-demographics, mental health and socio-economic status. (DOCX 66 kb)
