# Supplementary material for: The influence of caregiver depression on adolescent mental health outcomes: findings from refugee settlements in Uganda
Source: BMC Psychiatry. 2017 Dec 19;17:405. doi: 10.1186/s12888-017-1566-x (PMC5738231; doi:10.1186/s12888-017-1566-x)
Supplement: Supplementary file 1 — Adol final instrument. Adolescent quantitative assessment. Measures of socio-demographics, violence, psychosocial well-being and social support. (DOCX 113 kb) [file 12888_2017_1566_MOESM1_ESM.docx]

**Instrument for Uganda**

**Adolescent report**

1. Survey number:
2. Cluster Number:
3. Identifying information for child:
   1. Name:
   2. Phone Number:

Before proceeding, ensure that you have read the respondent the following:

1. Study introduction

Do not proceed until you have:

1. Obtained permission to speak to the adolescent respondent

2. Obtained informed consent from the caregiver respondent

3. Obtained informed consent from the adolescent respondent

**To be completed by interviewer:**

- 1. **Is the respondent male or female?**

1. Male
2. Female

| 1. **SECTION ONE** |
| --- |

**1.2How old are you now?**

_______ years old

(88) Don't Know

(99) No Response

**1.3What country were you born in?**

1. South Sudan**[🡪 1.5]**
2. Uganda**[🡪1.4]**

(2) Other (specify) _________________________**[🡪 1.4]**

- 1. **How many months have you lived in Uganda? ____________**

**1.6 How many months have you lived in this Kiryandongo? _______________**

**1.7What is your current marital status? Are you…**

(1) Single [🡪 1.8]

(2) Married [🡪 1.7a]

(3) Widowed [🡪1.8]

(4) Divorced [🡪 1.8]

(5) In a relationship [🡪 1.7b] or

(6) Something else? (specify) _________ [🡪 1.8]

(88) Don’t know [🡪1.8]

(99) No response [🡪1.8]

**1.7a At what age did you get married? _ _**

**1.7b Are you currently living with your partner?**

(1) No

(0) Yes

**1.8 Do you have any living biological children of your own? Children may be living elsewhere.**

| (1) Yes**[🡪1.8a]** | (0) No**[🡪 1.9]** | (88)DK **[🡪 1.9]** | (99)NR**[🡪 1.9]** |
| --- | --- | --- | --- |

**1.8a How many children do you have? __ __** (99) No response

| ***READ OUT LOUD:***  Now I’d like to ask some questions about your biological parents, your natural parents who gave birth to you and the people you live with in your home. |
| --- |

**1.9. Is your biological mother alive?**

| (1) Yes**[🡪 1.9a]** | (2) No**[🡪1.10]** | (88)Don’t know**[🡪1.10]** | (99)NR**[🡪1.10]** |
| --- | --- | --- | --- |

**1.9a Does your biological mother live in the same household with you?**

| (1) Yes | (2) No | (88) Don’t know | (99) No response |
| --- | --- | --- | --- |

**1.10Is your biological father alive?**

| (1) Yes**[🡪 1.10a]** | (2) No**[🡪1.11]** | (88)Don’t know**[🡪1.11]** | (99)NR **[🡪1.11]** |
| --- | --- | --- | --- |

**1.10a Does your biological father in the same household with you?**

| (1) Yes | (2) No | (88)Don’t know | (99)No response |
| --- | --- | --- | --- |

**1.11 Who is directly responsible for your care? By being responsible for your care, I mean the person who provides food, clothing, and any other basic needs and also provides emotional care.**

***(DO NOT READ LIST. SELECTUP TO TWO RESPONSES.)***

1. Biological Mother
2. Biological Father
3. Live alone
4. Husband or Wife
5. My children
6. Non relative guardian/adoptive parents
7. Grandparent
8. Mother or Father’s brother or sister (Aunt/Uncle)
9. Step-Parent
10. Brother or sister
11. Other relative
12. Friend
13. Boss
14. Others (*specify*)________________________________________

88. Don’t Know

99. No response

**1.12 Who are all the people living with you in the house?When I say“living here” I mean “usually sleep under this roof”.**

***(Do not read. Listen to answer and SELECT all responses mentioned)***

1. Biological Mother
2. Biological Father
3. Live alone
4. Husband or Wife
5. My children
6. Non relative guardian/adoptive parents
7. Grandparent
8. Mother or Father’s brother or sister (Aunt/Uncle)
9. Step-Parent
10. Brother or sister
11. Other relative
12. Friend
13. Boss
14. Others (*specify*)________________________________________

88. Don’t Know

99. No response

**1.12a What is the total number of the people living in this household? _________**

| ***READ OUT LOUD***  Now I’d like to ask you some questions about the schooling you have received, as well as some of your experiences in school. |
| --- |

**1.13 Have you everattended school?**

| 1 Yes **[🡪 1.14]** | 2 No **[🡪1.13a]** | 88 DK**[🡪1.14]** | 99 NR**[🡪1.14]** |
| --- | --- | --- | --- |

**1.13a. Why have you never attended school (*Do not read list. Listen and SELECT all that apply. wait for answer and then ask -* “Anything else?”)**

1. I was sick

2. I had to care for a sick relative

3. I had to work

4. I had to go and stay with family/friends in another area

5. I am not treated well at school

6. No money for fees, uniform, books, or transportation

7. I was pregnant

8. I made a girl pregnant

9. I did not want to go

10. The school is too far

11. School not open.

12. My parents did not want me to go to school

13. Disability

14. Other (*specify*): ______________________________________________

88. Don’t know

99. No response

**1.14 What is the highest level of school you have completed? *(DO NOT READ. SELECT ONLY ONE)________***

| 1 | Pre-primary Nursery / Kindergarten |
| --- | --- |
| 2 | Some Primary |
| 3 | All Primary |
| 4 | Some Secondary School |
| 5 | All Secondary School |
| 6 | Vocational Training |
| 7 | University |
| 8 | Other (*specify*) |
| 88 | Don’t know |
| 99 | No response |

**1.15 Have you attended primary or secondary school in term 2 or term 3?**

| 1 Yes **[🡪 1.15c]** | 2 No **[🡪1.15a]** | 88 DK **[🡪1.15c]** | 99 NR **[🡪1.15c]** |
| --- | --- | --- | --- |

**1.15a. What were the reasons you did not attend primary or secondary school in the last term 2 or term 3 (*Do not read list. Listen and SELECT all that apply. wait for answer and then ask -* “Anything else?”)**

1. I was sick

2. I had to care for a sick relative

3. I had to work

4. I had to go and stay with family/friends in another area

5. I am not treated well at school

6. No money for fees, uniform, books, or transportation

7. I was pregnant

8. I made a girl pregnant

9. I did not want to go

10. The school is too far

11. School not open.

12. My parents did not want me to go to school

13. Disability

14. Other (*specify*): ______________________________________________

88. Don’t know

99. No response

**1.15c How often did you attend school in the past term 2 or term 3. Did you attend…**

1. Every day

2. Most days

3. Some days or

4. Few days?

**1.16 How much of the time do you feel safe at school? Do you feel safe at school…**

[0] All of the time

[1] Most of the time

[2] Some of the time or

[3] None of the time?

**1.17 How much of the time do you feel safe at home? Do you feel safe at home…**

[0] All of the time

[1] Most of the time

[2] Some of the time or

[3] None of the time?

**1.18 Do you have your own attestation card?**

[0] Yes

[1] No

| **SECTION TWO** |
| --- |

| ***READ OUT LOUD***  I am going to read you a list of sentences that describe how people feel. Please respond whether this is “Not True or Hardly Ever True” or “Somewhat True or Sometimes True” or “Very True or Often True” for you in **the past three months.** READ RESPONSES AFTER EACH QUESTION |
| --- |

**2.1 In the past three months, you have gotten really frightened for no reason at all. Is this…**

1. Not True or Hardly Ever True
2. Somewhat True or Sometimes True or
3. Very True or Often True?

**2.2 In the past three months, you have been afraid to be alone in the house. Is this…**

1. Not True or Hardly Ever True
2. Somewhat True or Sometimes True or
3. Very True or Often True?

**2.3 In the past three months, people have told you that you worry too much. Is this…**

1. Not True or Hardly Ever True
2. Somewhat True or Sometimes True or
3. Very True or Often True?

**2.4 In the past three months, you have been scared to go to school. Is this…**

1. Not True or Hardly Ever True
2. Somewhat True or Sometimes True or
3. Very True or Often True?

**2.5 In the past three months, you have been shy. Is this…**

1. Not True or Hardly Ever True
2. Somewhat True or Sometimes True or
3. Very True or Often True?

| ***READ OUT LOUD***  Please say if the following statements are true: none of the time, some of the time, most of the time or all of the time. |
| --- |

**2.6 You think you are doing pretty well. Is this true…**

1. None of the time
2. Some of the time
3. Most of the time or
4. All of the time?

**2.7 You can think of many ways to get the things in life that are most important to you. Is this true…**

1. None of the time
2. Some of the time
3. Most of the time or
4. All of the time?

**2.8 You are doing just as well as other kids your age. Is this true…**

1. None of the time
2. Some of the time
3. Most of the time or
4. All of the time?

**2.9 When you have a problem, you can come up with lots of ways to solve it. Is this true…**

1. None of the time
2. Some of the time
3. Most of the time or
4. All of the time?

**2.10 You think the things you have done in the past will help you in the future. Is this true…**

1. None of the time
2. Some of the time
3. Most of the time or
4. All of the time?

**2.11 Even when others want to quit, you know that you can find ways to solve the problem. Is this true…**

1. None of the time
2. Some of the time
3. Most of the time or
4. All of the time?

| ***READ OUT LOUD***  Are the following statements true, sometimes true, or not true about how you have been feeling in the past two weeks? |
| --- |

**2.39 In the past two weeks, you felt miserable or unhappy. Is this…**

(0) True

(1) Sometimes true, or

(2) Not true?

**2.40 In the past two weeks, you didn’t enjoy anything at all. Is this…**

(0) True

(1) Sometimes true, or

(2) Not true?

**2.41 In the past two weeks, you felt so tired that you just sat around and did nothing. Is this…**

(0) True

(1) Sometimes true, or

(2) Not true?

**2.42 In the past two weeks, you were very restless. Is this…**

(0) True

(1) Sometimes true, or

(2) Not true?

**2.43 In the past two weeks, you felt that you are no good anymore. Is this…**

(0) True

(1) Sometimes true, or

(2) Not true?

**2.44 In the past two weeks, you cried a lot. Is this…**

(0) True

(1) Sometimes true, or

(2) Not true?

**2.45 In the past two weeks, you found it hard to think properly or concentrate. Is this…**

(0) True

(1) Sometimes true, or

(2) Not true?

**2.46 In the past two weeks, you hated yourself. Is this…**

(0) True

(1) Sometimes true, or

(2) Not true?

**2.47 In the past two weeks you were a bad person. Is this…**

(0) True

(1) Sometimes true, or

(2) Not true?

**2.48 In the past two weeks, you felt lonely. Is this…**

(0) True

(1) Sometimes true, or

(2) Not true?

**2.49 In the past two, weeks you thought nobody really loves you. Is this..**

(0) True

(1) Sometimes true, or

(2) Not true?

**2.50 In the past two weeks, you thought you could never be as good as other kids. Is this…**

(0) True

(1) Sometimes true, or

(2) Not true?

**2.51 In the past two weeks, you did everything wrong. Is this…**

(0) True

(1) Sometimes true, or

(2) Not true?

| **SECTION 3** |
| --- |

| ***READ OUT LOUD***  We want to find out about experiences that happen to young people at home or inside the family. We want to find out about the things that adults sometimes do to children and adolescents that may hurt or make them feel uncomfortable, upset or scared in their home. These questions may seem strange or hard to answer. Please try to answer them as best you can. This is not a test. There is not right or wrong answer, just say what you remember happened to you. If at any point you feel too uncomfortable to continue you can stop. We want to ask you about things that have happened to you since the last South Sudanese New Year. We will not ask about who in the household may have done any of these things, just whether it was an adult, a child or both.  If you want to get help about any of the things we ask about, talk to the person who gave this interview to you. Unless you tell us you want to talk, no one will ever know that the answers that you give are about you |
| --- |

**3.1 Has anyone in your home ever used drugs and/or alcohol and then behaved in a way that frightened you?**

(1) Yes**[🡪 3.1a]**

(0) No**[🡪 3.2]**

**3.1a Since the last South Sudan Independence Day, has this happened…**

(2) Many times

(1) Sometimes or

(0) Not since last South Sudanese Independence Day?

**3.2 Have you ever seen adults in your home shouting and yelling at each other (arguing) in a way that frightened you?**

(1) Yes **[🡪 3.2a]**

(0) No **[🡪 3.3]**

**3.2a Since the last South Sudan Independence Day, has this happened…**

(2) Many times

(1) Sometimes or

(0) Not since last South Sudanese Independence Day?

**3.3 Have you seen adults in your home hit, kick, slap, punch each other or hurt each other physically in other ways?**

(1) Yes**[🡪 3.3a]**

(0) No**[🡪 3.4]**

**3.3a Since the last South Sudan Independence Day, has this happened…**

(2) Many times

(1) Sometimes or

(0) Not since last South Sudanese Independence Day?

**3.4 Have you ever seen anyone in your home use knives, guns, sticks, rocks or other things to hurt or scare someone else inside the home?**

(1) Yes **[🡪 3.4a]**

(0) No**[🡪 next instruction box]**

**3.4a Since the last South Sudan Independence Day, has this happened…**

(2) Many times

(1) Sometimes or

(0) Not since last South Sudanese Independence Day?

| READ OUT LOUD  Sometimes, when children and adolescents are growing up, people say or do things to make the child or adolescent feel embarrassed, ashamed or bad. We’re going to ask you about your family and people living in your home. This may be natural parents, grandparents, step parents, adoptive parents, caregivers, aunts and uncles, or brothers, sister or cousins. |
| --- |

3.5 Has anyone in your family or living in your home ever screamed at you very loudly and aggressively?

(1) Yes **[🡪 3.5a]**

(0) No**[🡪 3.6]**

**3.5a Since the last South Sudan Independence Day, has this happened…**

(2) Many times

(1) Sometimes or

(0) Not since last South Sudanese Independence Day?

**3.6 Has anyone in your family or living in your home ever called you names, said mean things or cursed you?**

(1) Yes **[🡪 3.6a]**

(0) No**[🡪 3.7]**

**3.6a Since the last South Sudan Independence Day, has this happened…**

(2) Many times

(1) Sometimes or

(0) Not since last South Sudanese Independence Day?

**3.7 Has anyone in your family or living in your home ever said that they wished you were dead/ had never been born?**

(1) Yes **[🡪 3.7a]**

(0) No**[🡪 3.8]**

**3.7a Since the last South Sudan Independence Day, has this happened…**

(2) Many times

(1) Sometimes or

(0) Not since last South Sudanese Independence Day?

**3.8 Has anyone in your family or living in your home ever threatened to leave you forever or abandon you?**

(1) Yes **[🡪 3.8a]**

(0) No**[🡪 3.9]**

**3.8a Since the last South Sudan Independence Day, has this happened…**

(2) Many times

(1) Sometimes or

(0) Not since last South Sudanese Independence Day?

**3.9 Has anyone in your family or living in your home ever threatened to hurt or kill you, including invoking evil spirits against you?**

(1) Yes **[🡪 3.9a]**

(0) No**[🡪 3.10]**

**3.9a Since the last South Sudan Independence Day, has this happened…**

(2) Many times

(1) Sometimes or

(0) Not since last South Sudanese Independence Day?

| ***READ OUT LOUD***  Sometimes people that live in the same home as children and adolescents can hurt them physically. Thinking about yourself, in the past year, has anyone in your home (for example natural parents/ grandparents/ stepparents/adoptive parents/ caregivers/aunts and uncles/ older brother or sisters or cousins) done something such as: |
| --- |

3.10 Has anyone in your family or living in your home ever pushed, grabbed or kicked you?

(1) Yes **[🡪 3.10a]**

(0) No**[🡪 3.11]**

**3.10a Since the last South Sudan Independence Day, has this happened…**

(2) Many times

(1) Sometimes or

(0) Not since last South Sudanese Independence Day?

**3.10b Was it by an…**

(1) Adult

(2) Another child or adolescent, or

(3) Both?

**3.11 Has anyone in your family or living in your home ever hit, beat or spanked you with a hand?**

(1) Yes **[🡪 3.11a]**

(0) No**[🡪 3.12]**

**3.11a Since the last South Sudan Independence Day, has this happened…**

(2) Many times

(1) Sometimes or

(0) Not since last South Sudanese Independence Day?

**3.11b Was it by an…**

(1) Adult

(2) Another child or adolescent, or

(3) Both?

**3.15 Has anyone in your family or living in your home ever threatened you with a knife or a gun**

(1) Yes **[🡪 3.15a]**

(0) No**[🡪 3.16]**

**3.15a Since the last South Sudan Independence Day, has this happened…**

(2) Many times

(1) Sometimes or

(0) Not since last South Sudanese Independence Day?

**3.15b Was it by an…**

(1) Adult

(2) Another child or adolescent, or

(3) Both?

| ***READ OUT LOUD***  I am going to ask you some questions about violence that might be difficult to answer. These answers are all confidential, and you do not have to answer any questions you do not want to. May I continue? |
| --- |

**3.16 Was there a time when you were physically forced to have sexual intercourse against your will?**

(1) Yes**[🡪3.16a]**

(0) No **[🡪3.17]**

**3.16a How many times has this happened since the last South Sudanese Independence Day? _______________**

**3.16b Did you tell anyone about this most recent incident?**

(0) Yes**[🡪3.16d]**

(1) No **[🡪3.16c]**

**3.16c Can you tell me any reasons why you didn’t tell anyone?**

**[DO NOT READ OUT LOUD, SELECT ALL THAT APPLY]**

1. I was scared I was going to be abandoned
2. Financially dependent upon the abuser
3. I wasn’t aware that it was abuse
4. I didn’t know who to tell
5. I didn’t think I would be believed
6. I didn’t want to embarrass my family
7. The abuser threatened to hurt me or my family
8. I was given money or gifts not to tell anyone
9. I didn't want to get the abuser in trouble
10. Other____________________

**3.16d Who did you tell?**

**[DO NOT READ OUT LOUD, SELECT ALL THAT APPLY.**

**Probe: “Anyone Else?”]**

(1) Mother

(2) Father

(3) Other relative

(4) Friend

(5) Teacher/Principal

(6) Religious leader

(7) Health care provider / doctor / nurse

(8) Traditional healer

(9) Police/someone from security sector

(10) Counsellor

(11) Community Leader

(12) Other_________________________

**3.16e What, if any,services did you receive because of this most recent incident?**

i. Did you receive legal assistance?

Yes No Don’t know No response

ii. Did you receive material assistance?

Yes No Don’t know No response

iii. Did you receive safety and security support (i.e. safe house)

Yes No Don’t know No response

iv.Did you receive psychosocial counselling?

Yes No Don’t know No response

v.Did you receive medical assistance?

Yes No Don’t know No response

vi.Did you receive individual case management, i.e. home visits and follow up?

Yes No Don’t know No response

**3.17 Was there a time when you were persuaded or pressured to have sexual intercourse against your will?**

(1) Yes**[🡪3.17a]**

(0) No **[🡪3.18]**

**3.17a How many times has this happened since the last South Sudanese New Year? _______________**

**3.17b Did you tell anyone about this most recent incident?**

(0) Yes**[🡪3.17d]**

(1) No **[🡪3.17c]**

**3.17c Can you tell me any reasons why you didn’t tell anyone?**

**[DO NOT READ OUT LOUD, SELECT ALL THAT APPLY]**

1. I was scared I was going to be abandoned
2. Financially dependent upon the abuser
3. I wasn’t aware that it was abuse
4. I didn’t know who to tell
5. I didn’t think I would be believed
6. I didn’t want to embarrass my family
7. The abuser threatened to hurt me or my family
8. I was given money or gifts not to tell anyone
9. I didn't want to get the abuser in trouble
10. Other____________________

**3.17d Who did you tell? [DO NOT READ OUT LOUD, SELECT ALL THAT APPLY]**

*Probe – anyone else?*)

(1) Mother

(2) Father

(3) Other relative

(4) Friend

(5) Teacher/Principal

(6) Religious leader

(7) Health care provider (such as a doctor or nurse)

(8) Traditional healer

(9) Police/someone from security sector

(10) Counsellor

(11) Community Leader

(12) Other_________________________

**3.17e What, if any, services did you receive because of this most recent incident?**

i. Did you receive legal assistance?

Yes No Don’t know No response

ii. Did you receive material assistance?

Yes No Don’t know No response

iii. Did you receive safety and security support (i.e. safe house)

Yes No Don’t know No response

iv. Did you receive psychosocial counselling?

Yes No Don’t know No response

v. Did you receive medical assistance?

Yes No Don’t know No response

vi. Did you receive individual case management, i.e. home visits and follow up?

Yes No Don’t know No response

**3.19 If a friend or someone you know had one of these experiences (forced sex or sexual abuse), who would you feel comfortable seeking help from? Do not say a person’s name. Please tell me your relationship to them. [DO NOT READ. CAN SELECT MORE THAN ONE]**

(1) Mother

(2) Father

(3) Other relative (Older brother, older sister)

(4) Friend

(5) Teacher/Principal

(6) Religious leader

(7) Health care provider (such as a doctor or nurse)

(8) Traditional healer

(9) Police/someone from security sector

(10) Counsellor

(11) Community Leader

(12) Other – who?_________________________

**3.20 How comfortable would you feel saying no to a partner or spouse who wanted to have sexual intercourse? *(Read all and SELECT one.)***

1. It is easy to say no

2. Sometimes it is easy to say no

3. Sometimes it is not easy to say no

4. It is not easy to say no

**3.21 How comfortable would you feel saying no to a respected adult in your family, other than your spouse, who wanted to have sexual intercourse? *(Read all and SELECT one.)***

1. It is easy to say no

2. Sometimes it is easy to say no

3. Sometimes it is not easy to say no

4. It is not easy to say no

**3.22 How comfortable would you feel saying no to a respected adult from your community who wanted to have sexual intercourse? *(Read all and SELECT one.)***

1. It is easy to say no

2. Sometimes it is easy to say no

3. Sometimes it is not easy to say no

4. It is not easy to say no

**3.24 During the past term, on how many days did you not go to school because you felt you would be unsafe at school or on your way to or from school?**

**(DO NOT READ OUTLOUD. CHOOSE ONE.)**

1. 0 days
2. 1 day
3. 2 or 3 days
4. 4 or 5 days
5. 6 or more days
6. Did not go to school in the past term (for other reason)

**3.25 During the past term, have you been hit, pushed, kicked or shoved on school property?**

1. Yes**[🡪 3.25a]**
2. No **[**🡪 **3.26]**

**3.26 Has someone threatened or injured you with a weapon such as a gun, knife, or stick on school property?**

1. Yes

0. No

**3.27 Have you been screamed or yelled at very loudly or aggressively at school?**

1. Yes**[🡪3.27a]**

0. No **[🡪3.28]**

**3.28 Has a teacher ever punished you by hitting or beating you?**

1. Yes

0. No

**3.29 During the past 30 days, on how many days did you avoid certain areas in the settlement as you felt you would be unsafe? [DO NOT READ OUT LOUD. SELECT ONE]**

1. 0 days
2. 1 day
3. 2 or 3 days
4. 4 or 5 days
5. 6 or more days

**3.30 Have you been hit, pushed, kicked or shoved in a public area of the settlement, apart from at school?**

(1) Yes

(0) No

**3.31 Has someone threatened or injured you with a weapon such as a gun, knife, or stick in a public area of the settlement, apart from at school?**

1. Yes
2. No

**3.33 Has a teacher or principal offered you money, gifts, food, shelter, or a better grade in school if you had sex with him/her?**

(1) Yes

(0) No

**3.34 Have you had sexual intercourse with a teacher or principal because you hoped to receive money, gifts, food, shelter, or a better grade in school?**

(1) Yes

(0) No

**3.35 Has someone other than a teacher or principal offered you money, gifts, food, services, or shelter if you had sex with him/her?**

(1) Yes

(0) No

**3.36 Have you had sexual intercourse with someone other than a teacher or principal because you hoped to receive money, gifts, food, services or shelter?**

(1) Yes**[🡪3.36a]**

(0) No **[🡪Next Section]**

**3.36a Who did you have sex with because you hoped to get money, gifts, food, services or shelter? I am not asking for any names. I am asking for the person’s relationship to you. *[DO NOT READ OUT LOUD. SELECT ALL THAT APPLY. ASK QUESTION, WAIT FOR ANSWERS AND THEN ASK: “Anyone else?”]***

1. Boyfriend / girlfriend
2. Husband/ wife
3. Sugar daddy/sugar mommy
4. Friend
5. Father
6. Brother
7. Other male relative
8. Family friend/lodger
9. Man/boy from neighborhood
10. Religious leader
11. Manager/Foreman/Employer
12. Stepfather/Mother’s boyfriend
13. Stranger
14. Recent acquaintance
15. Humanitarian worker
16. Other__________________

| **SECTION FOUR** |
| --- |

**4.1a In the past week, have you felt unsafe in your home?**

[1] Yes

[0] No

**4.1b In the past week, have you felt unsafe at school?**

[1] Yes

[0] No

[2] Not applicable (do not go to school)

**4.1c In the past week, have you felt unsafe at the market, or other public spaces in the settlement?**

[1] Yes

[0] No

**4.1d In the past week, have you felt unsafe on the way to or from school?**

[1] Yes

[0] No

[2] Not applicable (do not go to school)

**4.1e In the past week, have you felt unsafe on the way to or from market, or other public spaces in the settlement?**

[1] Yes

[0] No

**4.1f In the past week, have you felt unsafe at work?**

[1] Yes

[0] No

[2] Not applicable (do not work)

**4.1g In the past week, have you felt unsafe on the way to work?**

[1] Yes

[0] No

[2] Not applicable (do not work)

**4.2 Are there any other places you have felt unsafe in the past week?**

[1] Yes [🡪4.2a]

[0] No [🡪4.3]

**4.2a Where is that place? [specify]** _________________________

**4.3 Have you ever been injured while walking around the settlement?**

[1] Yes [🡪 4.3a]

[0] No [🡪 4.4]

**4.3a Can you describe the injury?**

**4.4 Are there police or security officials in the settlement?**

[0] Yes [🡪4.4a]

[1] No [🡪Go to Section 5]

**4.4a If you have a problem, would they help you?**

[0] Yes

[1] No

[88] Don’t know

| **SECTION FIVE** |
| --- |

| ***READ OUT LOUD***  We know that young people are sometimes expected to help out with ‘heavy work’. Please tell me whether/how often you were expected to do the following in the last week: |
| --- |

**5.1 During the past week, did you do any kind of work for someone who is not a member of your household?**

1) Yes [🡪 5.1a]

0) No [🡪5.2]

**5.1a In the past week, about how many hours did you do this work for someone who is not a member of the household?**

__

**5.1b For pay in cash or goods?**

0) Yes, for pay (cash or goods)

1) No, unpaid

**5.2 During the past week, did you fetch water, or collect firewood for household use?**

1) Yes [🡪 5.2a]

0) No [🡪5.3]

**5.2a In the past week, about how many hours did you fetch water or collect firewood for household use?**

____

**5.3 During the past week, did you do any paid or unpaid work on a family farm or in a family business or selling goods in market?**

1) Yes [🡪 5.3a]

0) No [🡪5.4]

**5.3a During the past week, about how many hours did you do this work?**

_____

**5.4 During the past week, did you help with household chores such as shopping, cleaning, washing clothes, cooking, or caring for children, old or sick people?**

1) Yes [🡪 5.4a]

0) No [🡪5.5]

**4.5a During the past week, about how many hours did you do this work?**

**5.6 During the past week did you miss school to do any of this work? Yes/No**

**5.8 Since the last South Sudanese Independence Day, Have you worked or done any business that brought in money to help your family?**

(1) Yes **[🡪 5.8a]**

(0) No **[🡪 5.9]**

(88) Don’t know **[🡪 5.9]**

(99) No response **[🡪5.9]**

**5.8a. Who decides how the money will be used in your household?**

**DO NOT READ ALOUD. MARK ONLY ONE.**

1. Respondent

2. Spouse/partner

3. Parent/Caregiver

4. Other *(specify*) _______________

88. Don’t know

99. No response

**5.8 b. What is the money spent on?**

**DO NOT READ OUT LOUD. CAN SELECT MORE THAN ONE.**

1. School fees

2. School materials

3. Food

4. Health needs

5. Clothes, shoes and other personal items

6. Other (*specify*) _______________

88. Don’t know

99. No response

**5.9 Why do you work? [DO NOT READ LIST. CAN CHOOSE MORE THAN ONE]**

1. Supplement family income

2. Help pay family debt

3. Help in household enterprise

4. Learn skills

5. Schooling not useful for future

7. Cannot afford school fees

6. School is too far/ no school

8. Not interested in school

9. To temporarily replace someone unable to work

10. Other…(specify)

**5.10 Have you ever been injured or had an illness due to your work?**

(1) Yes

(0) No

| **SECTION SIX** |
| --- |

**6.1 Do you know of a place to go to if you have experienced violence or abuse?**

[0] Yes**[🡪6.1a]**

[1] No **[🡪6.2]**

[2] Don’t know**[🡪6.2]**

**6.1a What is that place?** _______________

**6.2. Do you know where to go if you have a health problem?**

[0] Yes**[🡪6.2a]**

[1] No **[🡪6.3]**

[2] Don’t know**[🡪6.3]**

**6.2a What is that place?**______________________

**6.3 Do you know where to go if you have a problem at school?**

[0] Yes**[🡪6.3a]**

[1] No **[🡪6.4]**

[2] Don’t know **[🡪6.4]**

[3] Not applicable (do not go to school) **[🡪6.4]**

**6.3a What is that place?** _______________

**6.4 Do you know where to go if you have a problem at home?**

[0] Yes [🡪6.4a]

[1] No [🡪6.5]

[2] Don’t know [🡪6.5]

**6.4a What is that place?** _______________

**6.5 Do you know where to go if you have a problem at work?**

[0] Yes[🡪6.5a]

[1] No [🡪6.6]

[2] Don’t know [🡪6.6]

[3] Not applicable (do not work) [🡪6.6]

**6.5a What is that place? __________ __________________**

**6.6 Have you ever heard of The Child Protection Committee or CPC?**

Yes [🡪6.7] No [🡪6.9] Don’t Know[🡪6.7] No Response[🡪6.7]

**6.7 What do you think is the role of Child Protection Committee?**

***(DO NOT READ LIST. CAN SELECT MORE THAN ONE RESPONSE. PROBE – “ANYTHING ELSE?”)***

1. Raise awareness on child rights/ advocacy for children in the community
2. Monitor child protection in the community/identify vulnerable children
3. Give advice to children, parents, and other community members
4. Refer cases to social workers
5. Protect children from violence and abuse
6. Teach children good behavior and give them advice
7. I don’t know
8. Other specify___________________________________

**6.8 Have you ever asked for help from The Child Protection Committee or CPC?**

Yes No Don’t Know No Response

**6.9 Have you ever heard about….**

**(Read the list below and let respondent indicate “yes” or “no”**

1. UNHCR

Yes No

1. World Food Program (WFP)

Yes No

1. TPO

Yes No

1. Save The Children

Yes No

1. Interaid

Yes No

1. Windletrust

Yes No

1. Danish Refugee Agency

Yes No

1. Real Medicine Foundation

Yes No

**6.10 Have you ever had any help from….? Read out the list below and let respondent indicate “yes” or “no”**

1. UNHCR

Yes No

1. World Food Program (WFP)

Yes No

1. TPO

Yes No

1. Save The Children

Yes No

1. Interaid

Yes No

1. Windletrust

Yes No

1. Danish Refugee Agency

Yes No

1. Real Medicine Foundation

Yes No

**6.11 If you have a complaint, are you aware of a place where you can go?**

(0) Yes**[🡪7.11a]**

(1) No **[🡪7.12]**

**6.11a Have you ever made a complaint?**

(1) Yes**[🡪7.11b]**

(2) No **[🡪7.12]**

- 1. **Have you ever wanted to take part in a group activity such as a football, volleyball, netball competition or drama in the settlement?**

[0] Yes [🡪6.12a]

[1] No [🡪6.13]

**6.12a Have you ever participated in a group activity such as a football, volleyball, netball competition or drama in the settlement?**

[0] Yes [🡪6.13]

[1] No [🡪6.12b]

**6.12b What is the main reason why you did not participate? DO NOT READ ALOUD. SELECT ONLY ONE.**

1. Did not know of any structured recreation activities
2. Did not have time to participate
3. I do not enjoy those types of activities
4. I experience discrimination if I go to those activities
5. Other (specify) _____________________
   1. **Are there places in this settlement that you can go to hang out with your friends?**

[0] Yes

[1] No

- 1. **Have you wanted to participate in an organized group, or committee specifically for or adolescents? Examples would be Youth Club, Church youth groups, youth forum, peer support groups.**

[0] Yes [🡪6.14a]

[1] No [🡪6.15]

**6.14a Have you participated in an organized group, committee specifically for or adolescents since the last South Sudanese Independence Day? Examples would be Youth Club, Church youth groups, youth forum, peer support groups.**

[0] Yes **[🡪6.15]**

[1] No **[🡪6.14b]**

**6.14b What is the main reason why you did not participate? DO NOT READ ALOUD. SELECT ONLY ONE.**

1. Did not know of any clubs or committees
2. Did not have time to participate
3. I do not enjoy those types of activities
4. I experience discrimination if I go to those activities
5. Other: specify _____________________

**6.15 Have you wanted to participate in any non-formal education in the settlement, for example, after-school activities?**

[0 Yes [🡪6.15a]

[1] No [🡪6.16]

**6.15a Have you participated in any non-formal education in the settlement since the last South Sudanese Independence Day?**

[0] Yes **[🡪6.16]**

[1] No **[🡪6.15b]**

**6.15bWhat is the main reason why you did not participate?DO NOT READ ALOUD. SELECT ONLY ONE.**

1. Did not know of any non-formal education activities
2. Did not have time to participate
3. I do not enjoy those types of activities
4. I experience discrimination if I go to those activities
5. Other….specify __________________

**6.16 Have you wanted to participate in any life skills trainingin the settlement?**

[0] Yes **[🡪6.16a]**

[1] No **[🡪Section 7]**

**6.16a. Have you participated in any life skills training in the settlement since the last South Sudanese Independence Day?**

[0] Yes [🡪Section 7]

[1] No [🡪6.16b]

**6.16bWhat is the main reason why you did not participate? DO NOT READ ALOUD. SELECT ONLY ONE.**

1. Did not know of any life skills training activities
2. Did not have time to participate
3. I do not enjoy those types of activities
4. I experience discrimination if I go to those activities
5. Other….specify__________________

| **SECTION SEVEN** |
| --- |

7.1 Sometimes, when parents or the people who take care of children are vexed by things that children do, they will beat children hard. In your view, when are parents right to beat their children?

1. If the child is disobedient

1) Yes 2) No 88) Don’t Know 99) No Response

1. If the child disagrees withthe parent

1) Yes 2) No 88) Don’t Know 99) No Response

1. If the child runs away from home

1) Yes 2) No 88) Don’t Know 99) No Response

1. If the child does not want to go to school

1) Yes 2) No 88) Don’t Know 99) No Response

1. If the child does not want to go to work

1) Yes 2) No 88) Don’t Know 99) No Response

1. If the child does not care for brothers and sisters

1) Yes 2) No 88) Don’t Know 99) No Response

1. If the child wets bed

1) Yes 2) No 88) Don’t Know 99) No Response

1. If the child steals

1) Yes 2) No 88) Don’t Know 99) No Response

1. If the child takes drugs or liquor

1) Yes 2) No 88) Don’t Know 99) No Response

1. If the child refuses to get married

1) Yes 2) No 88) Don’t Know 99) No Response

| **SECTION EIGHT** |
| --- |

| ***READ OUT LOUD***  We are interested in how you feel about the following statements. You can strongly disagree, disagree, agree or strongly agree with each of the statements. |
| --- |

**8.1 There is a special person who is around when I am in need**

(1) Strongly disagree

(2) Disagree

(3) Agree

(4) Strongly Agree

(9) Refused

**8.2 There is a special person with whom I can share my joys and sorrows.**

(1) Strongly disagree

(2) Disagree

(3) Agree

(4) Strongly Agree

(9) Refused

**8.3 My family really tries to help me.**

(1) Strongly disagree

(2) Disagree

(3) Agree

(4) Strongly Agree

(9) Refused

**8.4 I get the emotional help and support I need from my family.**

(1) Strongly disagree

(2) Disagree

(3) Agree

(4) Strongly Agree

(9) Refused

**8.5 I have a special person who is a real source of comfort for me.**

(1) Strongly disagree

(2) Disagree

(3) Agree

(4) Strongly Agree

(9) Refused

**8.6 My friends really try to help me.**

(1) Strongly disagree

(2) Disagree

(3) Agree

(4) Strongly Agree

(9) Refused

**8.7 I can count on my friends when things go wrong.**

(1) Strongly disagree

(2) Disagree

(3) Agree

(4) Strongly Agree

(9) Refused

**8.8 I can talk about my problems with my family**

(1) Strongly disagree

(2) Disagree

(3) Agree

(4) Strongly Agree

(9) Refused

**8.9 I have friends with whom I can share my joys and sorrows.**

(1) Strongly disagree

(2) Disagree

(3) Agree

(4) Strongly Agree

(9) Refused

**8.10 There is a special person in my life who cares about my feelings.**

(1) Strongly disagree

(2) Disagree

(3) Agree

(4) Strongly Agree

(9) Refused

**8.11 My family is willing to help me make decisions.**

(1) Strongly disagree

(2) Disagree

(3) Agree

(4) Strongly Agree

(9) Refused

**8.12 I can talk about my problems with friends.**

(1) Strongly disagree

(2) Disagree

(3) Agree

(4) Strongly Agree

(9) Refused

| **END SURVEY** |
| --- |

**READ THIS:** “Thank you very much for participating in our survey. We really appreciate your time. I know this discussion might have been difficult for you. How are you feeling right now? Would you like to discuss any of these issues further with someone else?”
