# Supplementary material for: The influence of caregiver depression on adolescent mental health outcomes: findings from refugee settlements in Uganda
Source: BMC Psychiatry. 2017 Dec 19;17:405. doi: 10.1186/s12888-017-1566-x (PMC5738231; doi:10.1186/s12888-017-1566-x)
Supplement: Supplementary file 2 — Caregiver final instrument. Caregiver quantitative assessment. Measures of socio-demographics, mental health and socio-economic status. (DOCX 66 kb) [file 12888_2017_1566_MOESM2_ESM.docx]

**­­­­­Instrument for Uganda**

1. Survey number:
2. Cluster:
3. Identifying information for caregiver:
   1. Name:
   2. Phone Number:

| Before proceeding, ensure that you have read the respondent the following:   1. Study introduction   Do not proceed until you have:   1. Obtained permission to speak to the child respondent 2. Obtained informed consent from the caregiver respondent |
| --- |

**To be completed by interviewer:**

- 1. **Is the respondent male or female?**

1. Male
2. Female

| **SECTION ONE** |
| --- |

**1.2 How old are you now?**

___________years old

(88) Don't Know

(99) No Response

**1.3. What country were you born in?**

1. Uganda**[🡪1.5]**
2. Sudan/ South Sudan**[🡪1.4]**
3. Other (specify) _________________________**[🡪1.4]**
   1. **How many months have you lived in Uganda? ____________**

**1.7 What is the highest level of school you have completed?**

***(DO NOT READ LIST.* Choose *only one response.)***

1. Never attended school
2. Pre-primary Nursery / Kindergarten
3. Some Primary
4. All Primary
5. Some Secondary School
6. All Secondary School
7. Vocational Training
8. University
9. Other (*specify*): ______________________________________________

88. Don’t know

99. No response

**1.8 What is your current marital status? Are you…**

(1) Single [🡪 1.9]

(2) Married [🡪 1.8a]

(3) Widowed [🡪1.9]

(4) Divorced [🡪1.9]

(5) In a relationship [🡪 1.8a] or

(6) Something else? (Specify)__________________ [🡪 1.9]

(88) Don’t know [🡪1.9]

(99) No response [🡪1.9]

**1.8a Are you currently living with your partner?**

(0) No

(1) Yes

**1.9Do you have children under 18? ______________**

(0) No**[🡪 1.16]**

(1) Yes**[🡪 1.10]**

**1.10 How many children under the age of 18 do you have? ___________**

**1.11 How many of those children live in the same household as you? _____________**

**1.12 How many of those children live in a different household in this settlement? __________________**

- 1. **How many children have you had since living in this settlement? _______**

(0) Zero **[🡪 1.15]**

(1) 1 or more**[🡪1.14]**

**1.14 Of those children, how many were you able to obtain birth registration for? ________**

**1.15 How many of your children (born here or elsewhere) have *their own* refugee ID card or documentation? _______**

**1.16 Who are all of the people who live in this household?**

**(DO NOT READ LIST.CHOOSE ALL THAT APPLY. PROBE – ANYONE ELSE?)**

1. Biological Mother
2. Biological Father
3. Husband or Wife
4. Own child
5. Foster child
6. Other child (not niece or nephew)
7. Grandparent
8. Mother or Father’s brother or sister (Aunt/Uncle)
9. Step-Parent
10. Brother or sister
11. Niece / nephew
12. Other relative
13. Friend
14. Boss
15. Others (*specify*)________________________________________

88. Don’t Know

99. No response

- 1. **What is the total number of the people living in this household including yourself? _________**

| **SECTION TWO** |
| --- |

| ***READ OUT LOUD***  I will read you the following statements about rearing and educating children. For each question, please tell me if you “strongly disagree,” “disagree,” “agree,” or “strongly agree.” |
| --- |

**2.1 Children should be treated the same regardless of the differences among them. Do you…**

[1] Strongly disagree

[2] Disagree

[3] Agree or

[4] Strongly agree?

**2.2 Children should be allowed to disagree with adults. Do you…**

[1] Strongly disagree

[2] Disagree

[3] Agree or

[4] Strongly agree?

**2.4 Children have a right to their own point of view and should be allowed to express it. Do you…**

[1] Strongly disagree

[2] Disagree

[3] Agree or

[4] Strongly agree?

**2.7 A child’s ideas should be seriously considered in making family decisions. Do you…**

[1] Strongly disagree

[2] Disagree

[3] Agree or

[4] Strongly agree?

**2.8a Have you ever heard of a Child Protection Committee (CPC)?**

1) Yes **[🡪2.9]**  2) No **[🡪2.10]** 88) Don’t Know 99) No Response

**2.9 What do you think is the role of a Child Protection Committee?**

1. Raise awareness on child rights/ advocacy for children in the community

2. Monitor child protection in the community/identify vulnerable children

3. Give advice to children, parents, and other community members

4. Refer cases to social workers

5. Protect children from violence and abuse

6. Teach children good behavior and give them advice

7. I don’t know

8. Other specify___________________________________

**2.10 Sometimes, when parents or the people who take care of children are upset or angered by things that children do, they will beat children hard. I’m going to ask some questions and would like to know, in your view, when are parents right to beat their children?**

1. Should parents beat their children If the child is disobedient

1) Yes 2) No 88) Don’t Know 99) No Response

1. Should parents beat their children if the child disagrees with the parent

1) Yes 2) No 88) Don’t Know 99) No Response

1. Should parents beat their children if the child runs away from home

1) Yes 2) No 88) Don’t Know 99) No Response

1. Should parents beat their children if the child does not want to go to school

1) Yes 2) No 88) Don’t Know 99) No Response

1. Should parents beat their children if the child does not want to go to work

1) Yes 2) No 88) Don’t Know 99) No Response

1. Should parents beat their children if the child does not care for brothers and sisters

1) Yes 2) No 88) Don’t Know 99) No Response

1. Should parents beat their children if the child is engaged by adult in prostitution

1) Yes 2) No 88) Don’t Know 99) No Response

1. Should parents beat their children if the child wets bed

1) Yes 2) No 88) Don’t Know 99) No Response

1. Should parents beat their children if the child steals

1) Yes 2) No 88) Don’t Know 99) No Response

1. Should parents beat their children if the child takes drugs or liquor

1) Yes 2) No 88) Don’t Know 99) No Response

1. Should parents beat their children if the child refuses to get married

1) Yes 2) No 88) Don’t Know 99) No Response

**2.11Do you report when you see or hear of children experiencing abuse at home or in the community?**

i) Yes **[🡪 2.12]**

ii) No**[🡪 2.13]**

iii) Not applicable – have not seen or heard of abuse **[ 🡪 SECTION 3]**

**2.12 Whom do you normally report to? Do not say a person’s name, just say their relationship or title. (DO NOT READ LIST. WAIT FOR ANSWER AND THEN ASK “ANYONE ELSE?”)**

1. Family member/close friend
2. Community elder/Chief/chairperson
3. Religious leader
4. Teacher
5. Social Workers/psychologists
6. NGO
7. TPO
8. Save the Children
9. Interaid
10. Child Protection Committee
11. Police
12. Another person (specify)________________________________

| **WHEN YOU FINISH 2.12🡪 SECTION 3** |
| --- |

**2.13 What are the reasons for not reporting? (DO NOT READ LIST, CAN CHOOSE MORE THAN ONE)**

1. Don’t know where or to whom
2. Don’t care, it’s not my business
3. Normally these thing happen here
4. Perpetrator is respected in my community
5. I know the perpetrator.
6. Fear
7. I want to warn the perpetrator first
8. In the past I reported but nothing happened to the perpetrator
9. Other – specify _____________________________________

| **Section Three** |
| --- |

**3.1 Have you worked in the past seven days?**

1. No**[🡪 3.2]**
2. Yes**[🡪 3.4]**

**3.2 Although you did not work in the last seven days, do you have any job or business?**

1. No
2. Yes

**3.3 Have you done any work since the last South Sudan Independence Day?**

1. No**[🡪 3.5]**
2. Yes **[🡪 3.4]**

**3.4Do you usually work regularly throughout the year, seasonally, or only once in a while?**

1. Regularly throughout the year
2. Seasonally/ part of the year
3. Once in a while

**3.5 What is your main source of income?**

1. Farming
2. Wages
3. Business activities (i.e. selling products)
4. Selling food from WFP
5. Cash from international organization
6. Money from family member or friend
7. No income
8. Other [specify] ______________

**3.5a Do you receive any money from family members or friends not living in your home?**

(0) No**[🡪 3.6]**

(1) Yes **[🡪 3.5b]**

**3.5b Does this family member or friends live outside the camp?**

(0) No

(1) Yes

**3.6 Who in the household provides income for the household?**

**(DO NOT READ LIST, CAN CHOOSE MORE THAN ONE)**

(0) Myself

(1) Partner

(2) Child

(3) Other relative, over age 18

(4) No income

**3.7 What is the main source of drinking water for members of your household? (DO NOT READ LIST, CAN CHOOSE MORE THAN ONE)**

(0) Piped water

(1) Public tap/ standpipe

(2) Well or borehole

(3) Rainwater

(4) Tanker/ trunk

(5) Purchased purified water

(6) Stream or river

(7) Other (specify)

**3.8 How many hours does it take to go, collect water and return to your household?**

_______ ~~hours~~

(88)Don’t know

(99) No response

**3.9 How many rooms are there in your living structure? ____________**

**3.10 How many of these rooms are used for sleeping? ____________**

**3.11a Do any members of this household own a watch?**

Yes No Don’t Know No Response

**3.11b Do any members of this household have a bicycle?**

Yes No Don’t Know No Response

**3.11c Do any members of this household own a cellphone?**

Yes No Don’t Know No Response

**3.11d Do you have a table in your household?**

Yes No Don’t Know No Response

**3.11e Do you have a cooking pot in your household?**

Yes No Don’t Know No Response

**3.11f Do you have a jerry can in your household?**

Yes No Don’t Know No Response

**3.11g Do you have a basin in your household?**

Yes No Don’t Know No Response

**3.11h Do you have a mat/ blanket in your household?**

Yes No Don’t Know No Response

**3.11i Do you have a chair or stool in your household?**

Yes No Don’t Know No Response

**3.11j Do you have a radio in your household?**

Yes No Don’t Know No Response

**3.11k Does this household own any livestock, herds, other farm animals, or poultry?**

Yes No Don’t Know No Response

**3.12 In the past 4 weeks, was there ever no food to eat of any kind in your house because of lack of resources to get food?**

1. No**[🡪 3.13]**
2. Yes **[🡪3.12a]**

**3.12a How many times has this happened?**

**(DO NOT READ LIST. CHOOSE ONLY ONE.)**

(1) 1-2 times

(2) 3-10 times

(3) More than 10 times

**3.13 In the past 4 weeks, did you or any household member go to sleep at night hungry because there was not enough food?**

1. No**[🡪 3.14]**
2. Yes **[🡪 3.13a]**

**3.13a How many times has this happened?**

**(DO NOT READ LIST. CHOOSE ONLY ONE.)**

(1) 1-2 times

(2) 3-10 times

(3) More than 10 times

**3.14 In the past 4 weeks, did you or any household member go a whole day and night without eating anything at all because there was not enough food?**

1. No**[🡪 3.15]**
2. Yes **[🡪 3.14a]**

**3.14a How many times has this happened?**

**(DO NOT READ LIST. CHOOSE ONLY ONE.)**

(1) 1-2 times

(2) 3-10 times

(3) More than 10 times

**3.15 How much of the time do the children in your care have enough to eat? Do they have enough to eat…**

(1) Most of the time

(2) Some of the time, or

(3) Rarely or never?

**3.16 In the last week, how many meals per day did the children in your care eat?**

**(DO NOT READ LIST. CHOOSE ONLY ONE.)**

1. One time
2. Two times
3. Three times or more

(88) Don’t know

(99) No response

**3.17 Was there a time since the last South Sudanese Independence Day that any of your children did not get the medical care for an injury or illness that he or she needed?**

[1] Yes**[🡪 3.17a]**

[0] No **[🡪 Section 4]**

**3.17a What are the reasons why they did not get care?**

**(DO NOT READ LIST. CHOOSE the answers that are closest to what the person says)**

1. The health center is too far away
2. The quality of services at the health center is low
3. They do not have the medications for my child’s illness at the center
4. I have previously experienced discrimination at the health center
5. I could not afford to take time off work to take my child to the health center
6. I went but I was told to wait and come back too many times
7. Other _________________

**Humanitarian Emergency Settings Perceived Needs Scale (HESPER):**

| **READ OUT LOUD**  I am going to ask you about the serious problems that you may currently be experiencing. We are interested in finding out what you think − a serious problem is a problem that you consider serious. There are no right or wrong answers. I am going to ask you about your own serious problems first. |
| --- |

**3.18 Do you have a serious problem because you do not have enough water that is safe for drinking or cooking?**

[1] It is a serious problem

[0] Not a serious problem

[99] No response/ does not know/ not applicable

**3.19 Do you have a serious problem with food? For example, because you do not have enough food, or good enough food, or because you are not able to cook food.**

[1] It is a serious problem

[0] Not a serious problem

[99] No response/ does not know/ not applicable

**3.21 Do you have a serious problem because you do not have easy and safe access to a clean toilet?**

[1] It is a serious problem

[0] Not a serious problem

[99] No response/ does not know/ not applicable

**3.23 Do you have a serious problem because you do not have enough, or good enough, clothes, shoes, bedding or blankets?**

[1] It is a serious problem

[0] Not a serious problem

[99] No response/ does not know/ not applicable

**3.24 Do you have a serious problem because you do not have enough income, money or resources to live?**

[1] It is a serious problem

[0] Not a serious problem

[99] No response/ does not know/ not applicable

**3.25 Do you have a serious problem with your physical health? For example, because you have a physical illness, injury or disability.**

[1] It is a serious problem

[0] Not a serious problem

[99] No response/ does not know/ not applicable

**3.26 For men: Do you have a serious problem because you are not able to get adequate health care for yourself? For example, treatment or medicines.**

**For women: Do you have a serious problem because you are not able to get adequate health care for yourself? For example, treatment or medicines, or health care during pregnancy or childbirth.**

[1] It is a serious problem

[0] Not a serious problem

[99] No response/ does not know/ not applicable

**3.27 Do you have a serious problem because you feel very distressed? For example, very upset, sad, worried, scared, or angry.**

[1] It is a serious problem

[0] Not a serious problem

[99] No response/ does not know/ not applicable

**3.28 Do you have a serious problem because you or your family are not safe or protected where you live now? For example, because of conflict, violence or crime in your community, city or village.**

[1] It is a serious problem

[0] Not a serious problem

[99] No response/ does not know/ not applicable

**3.29 Do you have a serious problem because your children are not in school, or are not getting a good enough education?**

[1] It is a serious problem

[0] Not a serious problem

[99] No response/ does not know/ not applicable

**3.30 Do you have a serious problem because in your situation it is difficult to care for family members who live with you? For example, young children in your family, or family members who are elderly, physically or mentally ill, or disabled.**

[1] It is a serious problem

[0] Not a serious problem

[99] No response/ does not know/ not applicable

**3.31 Do you have a serious problem because you are not getting enough support from people in your community? For example, emotional support or practical help.**

[1] It is a serious problem

[0] Not a serious problem

[99] No response/ does not know/ not applicable

**3.32 Do you have a serious problem because you are separated from family members?**

[1] It is a serious problem

[0] Not a serious problem

[99] No response/ does not know/ not applicable

| **READ OUT LOUD**  The next few questions refer to people in your community*(?), so please think about members of your community when answering these questions. |
| --- |

**3.39 Is there a serious problem in your community because of an inadequate system for law and justice, or because people do not know enough about their legal rights?**

[1] It is a serious problem

[0] Not a serious problem

[99] No response/ does not know/ not applicable

**3.40 Is there a serious problem for women in your community because of physical or sexual violence towards them, either in the community or in their homes?**

[1] It is a serious problem

[0] Not a serious problem

[99] No response/ does not know/ not applicable

**3.41 Is there a serious problem in your community because people drink a lot of alcohol, or use harmful drugs?**

[1] It is a serious problem

[0] Not a serious problem

[99] No response/ does not know/ not applicable

**3.42 Is there a serious problem in your community because people have a mental illness?**

[1] It is a serious problem

[0] Not a serious problem

[99] No response/ does not know/ not applicable

**3.43 Is there a serious problem in your community because there is not enough care for people who are on their own? For example, care for unaccompanied children, widows or elderly people, or unaccompanied people who have a physical or mental illness, or disability.**

[1] It is a serious problem

[0] Not a serious problem

[99] No response/ does not know/ not applicable

| **Section Four** |
| --- |

| ***READ OUT LOUD***  I am going to ask you about safety for children in the camp. By safety, I mean whether the child is physically safe, from injury or abuse, in the following places. I will ask you to strongly agree, agree, disagree or strongly disagree about the following statements. |
| --- |

**4.1 A child in this camp is safe in their school. Do you…**

[0] Strongly agree

[1] Agree

[2] Disagree or

[3] Strongly disagree?

**4.2 A child in this camp is safe on their way to and from school. Do you…**

[0] Strongly agree

[1] Agree

[2] Disagree or

[3] Strongly disagree?

**4.3 A child in this camp is safe at the market or other open places in the camp. Do you…**

[0] Strongly agree

[1] Agree

[2] Disagree or

[3] Strongly disagree?

**4.4 Are there any places in the camp where children are unsafe?**

[1] Yes **[🡪4.4a]**

[0] No **[🡪 5.4]**

**4.4a Where is that place? ____________________**

**4.5 Has your child ever been injured while walking around the camp?**

[1] Yes **[🡪 4.5a]**

[0] No **[🡪 4.6]**

**4.5a If yes, how were they injured?**

**(open ended?)**

**4.5b Did your child require medical attention due to the injury?**

[1] Yes

[0] No

- 1. **Are there police or security officials in the camp?**

[1] Yes **[🡪 4.6a]**

[0] No

**4.6a If you have a problem, would they help you?**

[0] Yes

[1] No

| **Section Five** |
| --- |

| ***READ OUT LOUD***  Please listen carefully. We would like to know how you have been feeling during the last week, including today. We will ask you a question and give you several options to choose from for your answer. Please listen to all the options before answering. Select the option that you think most closely applies to you. Remember that we want to know about present and recent complaints, not those that you had in the past. It is important that you try to answer ALL the questions. Thank you very much for your co-operation. |
| --- |

**5.1 In the past week, how often have you felt suddenly scared for no reason? Have you felt this way…**

(0) Never

(1) Sometimes

(2) Often or

(3) All the time?

- 1. **In the past week, how often have you felt fearful? Have you felt this way…**

(0) Never

(1) Sometimes

(2) Often or

(3) All the time?

- 1. **In the past week, how often have you felt faintness, dizziness or weakness? Have you felt this way…**

(0) Never

(1) Sometimes

(2) Often or

(3) All the time?

**5.4 In the past week, how often have you felt nervousness or shakiness inside? Have you felt this way…**

(0) Never

(1) Sometimes

(2) Often or

(3) All the time?

- 1. **In the past week, how often have you felt that your heart was pounding or racing? Have you felt this way…**

(0) Never

(1) Sometimes

(2) Often or

(3) All the time?

- 1. **In the past week, how often have you felt your body trembling? Have you felt this way…**

(0) Never

(1) Sometimes

(2) Often or

(3) All the time?

- 1. **In the past week, how often have you felt tense or keyed up? Have you felt this way…**

(0) Never

(1) Sometimes

(2) Often or

(3) All the time?

- 1. **In the past week, how often have you had headaches? Have you had headaches…**

(0) Never

(1) Sometimes

(2) Often or

(3) All the time?

- 1. **In the past week, how often have you had spells of terror or panic? Have you felt this way…**

(0) Never

(1) Sometimes

(2) Often or

(3) All the time?

- 1. **In the past week, how often have you felt restless, or like you can’t sit still? Have you felt this way…**

(0) Never

(1) Sometimes

(2) Often or

(3) All the time?

- 1. **In the past week, how often have you been feeling low in energy, or slowed down? Have you felt this way…**

(0) Never

(1) Sometimes

(2) Often or

(3) All the time?

- 1. **In the past week, how often have you blamed yourself for things? Have you felt this way…**

(0) Never

(1) Sometimes

(2) Often or

(3) All the time?

- 1. **In the past week, how often have you cried easily? Have you felt this way…**

(0) Never

(1) Sometimes

(2) Often or

(3) All the time?

- 1. **In the past week, how often have you felt a loss of sexual interest or pleasure? Have you felt this way…**

(0) Never

(1) Sometimes

(2) Often or

(3) All the time?

- 1. **In the past week, how often have you had a poor appetite? Have you felt this way…**

(0) Never

(1) Sometimes

(2) Often or

(3) All the time?

- 1. **In the past week, how often have you had difficulty falling asleep or staying asleep? Have you felt this way…**

(0) Never

(1) Sometimes

(2) Often or

(3) All the time?

- 1. **In the past week, how often have you felt hopeless about the future? Have you felt this way…**

(0) Never

(1) Sometimes

(2) Often or

(3) All the time?

- 1. **In the past week, how often have you felt sad? Have you felt this way…**

(0) Never

(1) Sometimes

(2) Often or

(3) All the time?

- 1. **In the past week, how often have you felt lonely? Have you felt this way…**

(0) Never

(1) Sometimes

(2) Often or

(3) All the time?

- 1. **In the past week, how often have you felt like you were being trapped or caught? Have you felt this way…**

(0) Never

(1) Sometimes

(2) Often or

(3) All the time?

- 1. **In the past week, how often have you felt like you were worrying too much about things? Have you felt this way…**

(0) Never

(1) Sometimes

(2) Often or

(3) All the time?

- 1. **In the past week, how often have you felt a lack of interest in things? Have you felt this way…**

(0) Never

(1) Sometimes

(2) Often or

(3) All the time?

- 1. **In the past week, how often have you felt like everything is an effort? Have you felt this way…**

(0) Never

(1) Sometimes

(2) Often or

(3) All the time?

- 1. **In the past week, how often have you felt worthless? Have you felt this way…**

(0) Never

(1) Sometimes

(2) Often or

(3) All the time?

| **END SURVEY** |
| --- |

“Thank you very much for participating in our survey. We really appreciate you time. I know this discussion might have been difficult for you. How are you feeling right now? Would you like to discuss any of these issues further with someone else? Would you like for someone to come to visit you and discuss any of the issues you talked about in your interview today?”

**(LIST OF SERVICES to the caregiver)**
